# A Loud Auditory Stimulus Overcomes Voluntary Movement Limitation in Cervical Dystonia

**DOI:** 10.1371/journal.pone.0046586

**Published:** 2012-10-16

**Authors:** Tereza Serranová, Robert Jech, Maria José Martí, Raluca Modreanu, Francesc Valldeoriola, Tomáš Sieger, Evžen Růžička, Josep Valls-Solé

**Affiliations:** 1 Department of Neurology and Centre of Clinical Neuroscience, 1st Medical Faculty, Charles University in Prague, Prague, Czech Republic; 2 Parkinson's Disease & Movement Disorders Unit, Hospital Clínic, Universitat de Barcelona, Centro de Investigación en Red de Enfermedades Neurodegenerativas (CIBERNED), Institut d'Investigacions Biomèdiques August Pi i Sunyer (IDIBAPS), Hospital Clínic, Barcelona, Spain; 3 Department of Cybernetics, Faculty of Electrical Engineering, Czech Technical University in Prague, Prague, Czech Republic; 4 EMG and Motor Control Unit, Neurology Department, Hospital Clíınic, Facultad de Medicina, Universitat de Barcelona, Institut d'Investigacions Biomèdiques August Pi i Sunyer (IDIBAPS), Hospital Clínic, Barcelona, Spain; Weill Cornell Medical College, United States of America

## Abstract

**Background:**

Patients with cervical dystonia (CD) present with an impaired performance of voluntary neck movements, which are usually slow and limited. We hypothesized that such abnormality could involve defective preparation for task execution. Therefore, we examined motor preparation in CD patients using the StartReact method. In this test, a startling auditory stimulus (SAS) is delivered unexpectedly at the time of the imperative signal (IS) in a reaction time task to cause a faster execution of the prepared motor programme. We expected that CD patients would show an abnormal StartReact phenomenon.

**Methods:**

Fifteen CD patients and 15 age matched control subjects (CS) were asked to perform a rotational movement (RM) to either side as quick as possible immediately after IS perception (a low intensity electrical stimulus to the II finger). In randomly interspersed test trials (25%) a 130 dB SAS was delivered simultaneously with the IS. We recorded RMs in the horizontal plane with a high speed video camera (2.38 ms per frame) in synchronization with the IS. The RM kinematic-parameters (latency, velocity, duration and amplitude) were analyzed using video-editing software and screen protractor. Patients were asked to rate the difficulty of their RMs in a numerical rating scale.

**Results:**

In control trials, CD patients executed slower RMs (repeated measures ANOVA, p<0.10^−5^), and reached a smaller final head position angle relative to the midline (p<0.05), than CS. In test trials, SAS improved all RMs in both groups (p<0.10^−14^). In addition, patients were more likely to reach beyond their baseline RM than CS (χ^2^, p<0.001) and rated their performance better than in control trials (t-test, p<0.01).

**Conclusion:**

We found improvement of kinematic parameters and subjective perception of motor performance in CD patients with StartReact testing. Our results suggest that CD patients reach an adequate level of motor preparation before task execution.

## Background

Dystonia is characterized by excessive involuntary movements that interfere with willed actions, leading to abnormal postures and unwanted muscle activity [1]. Patients with cervical dystonia (CD) present with an impaired performance of voluntary neck movements [2], which are usually limited, slow and most of the times also painful [2]–[5]. The mechanisms accounting for such limitation in voluntary movements are incompletely understood [6]–[8]. Apart from other factors, excessive co-contraction of agonist and antagonist muscles, a characteristic feature of dystonia, should lead to slowness of movement [6]. Co-contraction, like other disorders of motor control in dystonic patients, may be due to an abnormal configuration of the motor programme.

The execution of purposeful movements implies the activation of a motor plan, which should set appropriate motor structures to the adequate level of excitability in preparation for the performance [9], [10]. In simple reaction time tasks, subjects can fully prepare the motor programme before delivery of the imperative signal (IS) for fast execution of the pre-defined task [9]. If subjects in such condition are presented with a loud startling auditory stimulus (SAS) together with the IS, their reaction times become significantly shorter, reaching latencies typical of a startle reaction, but maintaining the structure of the motor programme [11], [12]. The effect, termed the StartReact phenomenon, has been examined in various tasks and conditions in healthy subjects [11]–[14]. From these research studies, it is clear that a key aspect for the phenomenon to take place is preparation of motor circuits. This should imply an enhancement of excitability along all the structures of the motor pathway leading to task execution, ranging from premotor brain areas to the alpha motoneurons at the spinal cord. The exact point at which the SAS activates the motor system to trigger task execution in the StartReact test is unknwon but it has been shown that the motor cortex is implied [15].

The StartReact phenomenon has been examined also in patients. Valldeoriola et al. (1998) found it absent in patients with progressive supranuclear palsy, a disease in which the startle reaction is abnormally reduced [16]. Conversely, Anzak et al. (2011) found that patients with Parkinson's disease improved their performance when they were presented with the StartReact test to execute a gripping force dynamometer task [17]. With this background in mind, we wanted to find out what was the behavior of patients with cervical dystonia in the StartReact test. We hypothesized that, because of disturbed integration of sensory inputs in motor programmes, patients with dystonia might not be able to reach an appropriate level of preparation at the time of task execution. There is indeed evidence for an abnormal excitability in brainstem, spinal cord and motor cortex in dystonia [18], [19]. In CD, abnormal findings have been reported in various neurophysiological tests involving subcortical pathways and their relation with descending control inputs [20]–[23]. Therefore, we expected to find abnormalities in the StartReact phenomenon, either decrease or absence of the effect or, alternatively, distortion of the intended movement in the presence of a SAS. Patients with CD were selected because of their known difficulty in performing willed head movements and the possibility to compare those towards and against the predominating dystonic thrust. We tested our hypothesis by examining the effects of SAS on horizontal head rotational movements (RM) in patients and healthy controls.

## Materials and Methods

### Subjects

The study was carried out in 15 patients with primary CD, 8 male and 7 female, with a mean age of 47.1 years (ranging from 22 to 66). They were selected for the study if they presented with predominantly rotational dystonia with limitation for neck movements. The diagnosis of CD was based on the presence of abnormal posture or movements of the head and neck. We excluded patients with secondary forms as well as those with suspected psychogenic CD. We also excluded patients with severe dystonic tremor, because of the expected difficulties in the assessment of relevant movement parameters. Severity of the disorder was assessed by using the Toronto Western Spasmodic Torticollis Rating Scale [24] for CD. Demographical and clinical data of CD patients are detailed in Table 1. Patients who were on regular botulinum toxin treatment were studied at least three months after they received the treatment. The study protocol was approved by the local Ethics Committee and all subjects gave their informed consent for conducting the study and video recording. Fifteen healthy age and gender matched subjects (46.3 (24–66) years, 6 male and 9 female) served as controls (CS).

**Table 1:** Clinical characteristics of patients (N = 15).

Disease duration (years)	16.1 (11.6)
BTX treatment duration (years)	10.6 (7.6)
TWSTRS total score (max. = 85)	31.1 (12.4)
Torticollis severity Scale (max. = 35)	15.9 (4.2)
Disability scale (max. = 30)	9.6 (6.5)
Pain scale (max. = 20)	5.6 (5.3)

Values are expressed as means, with the standard deviation within parenthesis.

TWSTRS, Toronto Western Spasmodic Torticollis Rating Scale; BTX, Botulinum Toxin.

### Stimuli

The IS was a weak electrical shock delivered to the second finger of the right hand through a pair of ring electrodes at an intensity 2 times the perception threshold. A verbal forewarning preceeded always the IS by a variable period of 1–2 seconds. The SAS, of an intensity of 130 dB (sound pressure level), was produced by the discharge of the coil of a magnetic stimulator on top of a metallic platform at a distance of approximately 2 m from the subject [11].

The IS was issued from an electromyograph Synergy (CareFusion, Surrey, London), prepared for sending out simultaneously with the electrical stimulus a trigger pulse to switch on a 5 V light emitting diode in all trials, and to activate the stimulator for the delivery of SAS in test trials. The diode was positioned such that it was visible in the video recordings (see below) to indicate time 0 for all videotaped events.

### Recording

**Figure 1:**
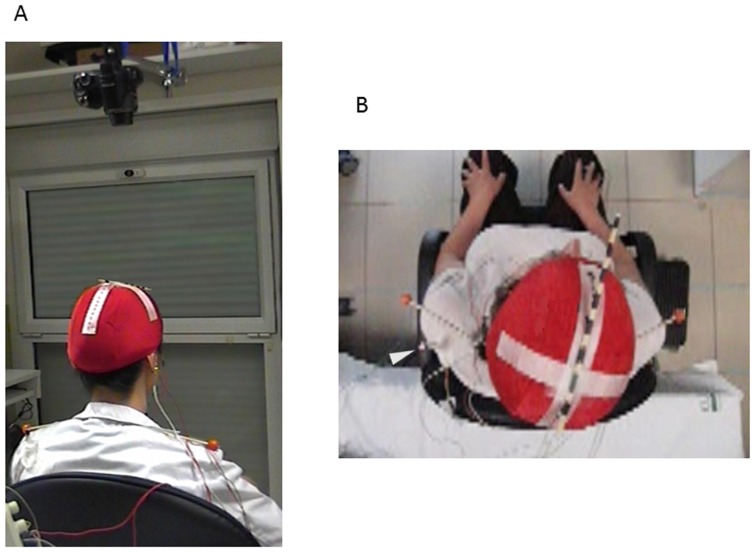
The experimental set up. A) Subjects were sitting comfortably under the focus of a high speed camera with a zenithal view. B) View from the camera. Note the stick marking the inion-to-nasion line and the shoulder markers. The subject is wearing the recording electrodes in the SCM and OO and the accelerometer attached to the chin.

A high speed video camera (Exilim FX25, Casio America, Inc.), which was able to record 420 frames per second, i.e. 2.38 ms per frame, was used to film head rotational movements (RM) from a zenithal position. The video camera was located 40 cm above the subjects head, oriented vertically downwards, focusing to the subjects' Cz. Subjects wore on their head an elastic cap with markers indicating the nasion-inion and the bi-auricular line. The nasion-inion line was further marked with a hatched stick attached to the cap (Figure 1). Another two markers were attached to the shoulders (acromions) for better visualization of shoulder movements and analysis of eventual head shifts relative to shoulders.

We also recorded the EMG activity using pairs of surface recording electrodes attached over the sternocleidomastoid (SCM) of both sides and the orbicularis oculi of the right side, as well as the head movement using an accelerometer attached to the lateral aspect of the chin. Band-pass frequency filters for EMG was 20 to 1000 Hz and for the accelerometer were 0.1 to 10 Hz. The sampling rate for signal storage was 2000 Hz. Recordings were done with a Synergy EMG machine (CareFusion, Surrrey, London).

### Procedure

Subjects were sitting on a chair where they were asked to keep a comfortable but steady upright posture in such a way that the mediosagittal plane relative to their pelvis was perpendicular to backrest. The subjects were left to adopt the head position in which they felt more comfortable while keeping the pelvis and trunk aligned with the armchair backrest. Patients were instructed not to compensate for their dystonic posture, which in most cases led to a deviation of the head towards one side (the ‘dystonic side’). Before starting data collection, subjects were requested to rotate their head to the extreme left and right positions at their own pace, in order to measure the individual's baseline range of head rotational movements (BRM), defined as the angle width between the two extreme positions. Then, they were asked the question: ‘How difficult is it for you to perform this movement?’, and they were instructed to describe the difficulty using a numerical rating scale where 0 was such difficulty that the subject perceived that ‘no movement was possible’ and 10 was no difficulty at all and, hence, the subject perception was that of ‘normal movement performance’.

Data collection began when subjects felt comfortable with the task after a few preliminary trials. They were instructed to be ready to react as quickly as possible at the perception of IS by rotating their head. The direction of head movement (either left or right sides, chosen pseudo-randomly at 50% chance) was clearly stated before forewarning but they were not informed on trial condition (whether or not there was going to be a SAS together with the IS). They were specifically instructed not to move their trunk during head RM. If such movement was observed during the experiment, the trial was repeated. A total of 15 trials were collected for each side per subject. Four of them contained the SAS (test trials). Trials of each condition (control and test) were presented in random order with an interval of 10–15 s between two consecutive trials. Randomly, we also applied the SAS with no warning in order to examine the startle reaction on its own. After the experiment, patients were asked the question: ‘Do you feel the same difficulties in performing the movement when the sound is present as when the sound is absent?’ If the answer indicated that there were differences between the two conditions, subjects were asked to rate separately the difficulties with performing the movement ‘when the sound was present’ and ‘when the sound was absent’, using the numerical rating scale in the same way as described above.

### Data analysis

The main outcome measure was the video-recordings for determination of head movement kinematics. For each trial, we identified three frames: Frame0, OnsetFrame and EndFrame. Frame0 was the first frame in which the light diode was seen to switch on. OnsetFrame was the frame in which the subject was seen to start the intended movement, i.e., the first frame with a detectable change in the hashed stick position in the intended direction of RM that was progressive in successive frames. EndFrame was the frame in which the movement reached its maximum in the intended direction of the RM.

**Figure 2:**
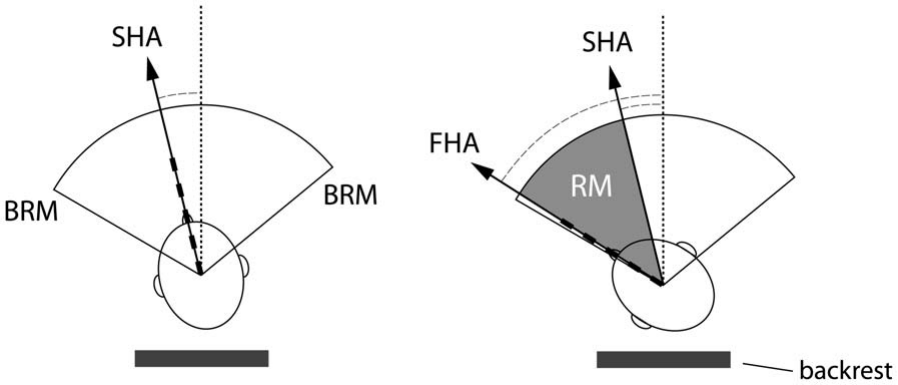
Schematic representation of the kinematic measures taken from the analysis of video-recordings. SHA = Starting head position angle; measured as the inion-to-nasion line angle with respect to the midline at frame0 BRM = Baseline range of head rotational movements, defined as the angular width between the two extreme positions reached in self-paced rotational movements towards each side. FHA = Final head position angle, measured as the inion-to-nasion line angle with respect to the midline at EndFrame. RM = Rotational movement. The scheme represents the RM amplitude, defined as the angular difference of the inion-to-nasion lines between OnsetFrame and EndFrame. Dotted line = midline See text for more details.

In each frame the head position indicated by the nasion-inion line was related to the midline, defined as a line plotted perpendicular to the armchair backrest. Angles between the nasion-inion line and the midline were measured in degrees using a goniometer software (MB-Ruler 5.0, Markus Bader, Iffezheim, Germany). The starting head position angle (SHA) was measured between the inion-to-nasion line and the midline at frame0, considering as positive value the deviation towards the side of the intended movement and a negative value the deviation towards the opposite side. We defined three parameters for each trial. RM latency, calculated in ms as the number of frames from Frame0 to OnsetFrame times 2.38 (the duration of each frame), RM duration, calculated in ms by multiplying the number of frames counted between OnsetFrame and EndFrame times 2.38, and RM amplitude, calculated in degrees by substracting the RM angle at the OnsetFrame from that at the EndFrame. Mean angular velocity was derived in each trial from the change in head position in degrees as a function of time [RMamplitude (°)/RMduration (s)]. We also determined the final, most extreme, head position angle (FHA) by measuring the RM angle at EndFrame with respect to the midline, regardless of the individual SHA. Figure 2 shows a schematic diagram of all measures. Trunk stability during head movements was assessed by measuring the angle between the bi-acromial line and a line parallel to the backrest in the same frames in which we measured RM. Trials in which this angle changed more than 5° were excluded from statistical analysis.

In the EMG recordings, we measured onset latency as the time elapsed between IS and the onset of EMG activity in the agonist SCM or, if there was background activity, at the point where a significant increase was noted (more than 50% of baseline) and was consistent for at least 50 ms. We also measured the EMG area using the automatic measurement feature of the electromyograph for the first 200 ms after onset latency. Since we were mainly interested in measuring the amount of co-contraction between antagonist SCM muscles, we calculated the ratio between SCM muscles by dividing the area measured in the agonist (contralateral to the direction of the requested movement) by that measured in the antagonist (ipsilateral to the direction of the requested movement). Data from the orbicularis oculi were used for monitoring the response to the startle and data from the accelerometer were used to assess the initial direction of the movement.

As all the parameters (RM latency, duration, velocity, relative amplitude, FHA) followed a normal distribution, parametric tests were applied. For normalization of data on RM amplitude, we expressed the values as percentages of the individual's BRM. As there was no a priori hypothesis which kinematic factor would be critical to reject the null hypothesis we entered all five kinematic parameters into a single multivariate general linear model (GLM) with repeated measures. Results were subsequently tested *post hoc* using univariate analyses of variance with repeated measures (ANOVA). Data from each trial were analyzed from the perspective of three factors: RM DIRECTION (right and left for CS, and ‘towards’ and ‘against’ the dystonic thrust for CD), experimental CONDITION (control, in which only the IS was presented, and test, in which the IS was presented together with the SAS) and subjects' GROUP (CS and CD). The χ^2^ test was used for analysis of nominal parameters. All statistical analyses were done with SPSS 14.0.1 software (Chicago, IL).

## Results

All subjects (CS and CD) were able to perform the task and complete the experiment with no difficulties although a few patients complained of neck pain and mild discomfort with repeating head movements (generally in RM against the dystonic thrust). We excluded a total of 15 trials because of significant trunk movement unnoticed during the experiment (3 in CS and 12 in CD, with no more than 2 in any single subject). As expected, because of the intrinsic limitation of neck movements in patients with cervical dystonia, we found statistically significant differences between groups in BRM, which mean value was 110.4° (SD = 20.3°) in CD and 139.0° (SD = 16.8°) in CS (ANOVA, between subjects factor: GROUP, F = 18.5, P<0.0001). When measured for each side separately relative to the midline, we found an effect of DIRECTION on the BRM in patients. This was significantly smaller against (50.6°, SD = 12.2°) than towards (59.8°, SD = 10.3°) dystonia (ANOVA, within subjects factor: DIRECTION, F = 12.7, P<0.01). No significant differences were found in BRM towards right (69.1°, SD = 9.4°) and left (69.9°, SD = 8.3°) sides in CS (ANOVA, within subjects factor: DIRECTION, F = 0.3, P = 0.6).

**Figure 3:**
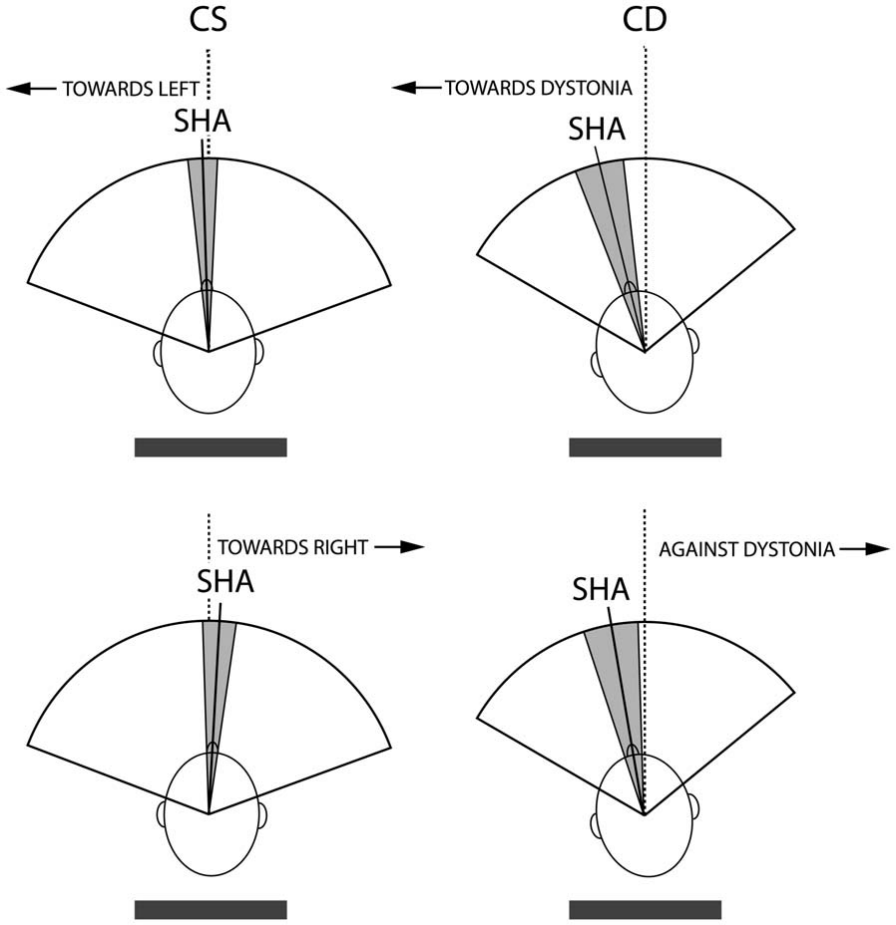
Schematic representation of data obtained in measuring the starting head angle. Data obtained in measuring the starting head angle (SHA) in control subjects (CS) and patients with cervical dystonia (CD) are shown for rotational movements (RMs) intended for left and right sides in CS and ‘towards’ and ‘against’ dystonia in CD.

Mean SHA was obtained by averaging control and test trials together, since it was determined at a time in which subjects did not know about the experimental condition. In CS, SHA showed a slight deviation towards the intended movement direction, while in patients, it showed a deviation towards the rotational component of the dystonia regardless of the intended movement direction (Figure 3). The statistical analysis showed that SHA was significantly different between CS and CD (ANOVA, between subjects factor: GROUP, F = 12.8, P<0.001). All subjects exhibited a response to the first SAS applied on its own, when subjects were not prepared to react (no IS and no warning). This caused a head flexion movement with no apparent differences between CS and CD. No RM was observed in those trials.

### Comparison between CS and CD patients

**Figure 4:**
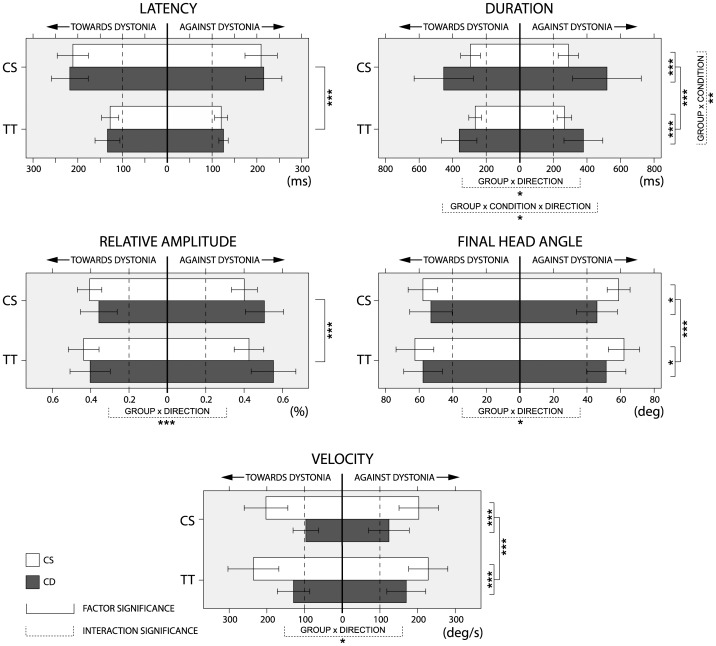
Schematic representation of data obtained in measuring the kinematic parameters of rotational head movements. Data on kinematic parameters of rotational head movements (RM) were extracted from high-speed video-recordings, in control subjects (CS) and patients with cervical dystonia (CD) in control and test trials. Data in CS are shown for right and left side RMs while data in CD are shown for RM ‘towards’ and ‘against’ the dystonic thrust. Significant differences are shown for factor (full lines) and interaction (dashed lines) according to the number of asterisks (* = P<0.05, ** = P<0.01, *** = P<0.001). The whiskers represent standard deviations. Note the similarity of the effects occurring in test trials in all kinematic parameters in both groups of subjects.

All parametric data extracted from video recordings in regard to control and test trials in CS and CD are summarized in Figure 4. The multivariate repeated analysis showed significant differences between CS and CD when all kinematic parameters were considered (between subjects factor: GROUP, F = 9.9, P<0.0001). Group differences were still significant even if direction of RM (interaction: GROUP x DIRECTION, F = 7.4, P<0.001) or condition (interaction: GROUP x CONDITION, F = 5.2, P<0.01) were considered. In the case of direction, differences were found in CD patients between RMs towards and against the dystonic thrust. In the case of condition, although the SAS presentation had a strong impact on kinematic parameters in both groups of subjects (within subject factor: CONDITION, F = 55.7, P<10^−12^), the effect seen in CD was significantly larger than in CS (interaction: GROUP x CONDITION, F = 5.2, P<0.01). To explore each of the parameters in more detail, a *post hoc* repeated measures ANOVA was performed separately for each variable (Figure 4).

### Latency

There was no significant effect of group on RM latency (between subject factor: GROUP, F = 0.5, P = 0.47). However, there was a strong effect of condition (within subject factor: CONDITION, F = 210.4, P<10^−14^), which consisted on a significantly earlier start in test trials than in control trials. The mean latency shortening was 87 ms (SD = 35 ms) in CD and 86 ms (SD = 33 ms) in CS. There was no significant effect of direction on latency, and there was no significant interaction between factors.

### Duration

There was a significant effect of group, which was due to a longer duration of RM in CD than in CS (between subject factor: GROUP, F = 17.0, P<0.001). The RM duration shortened in test trials in both groups of subjects (within subject factor: CONDITION, F = 22.4, P<0.0001). In addition, there was also a significant GROUP x CONDITION interaction (F = 9.2, P<0.01) implying that RM duration was affected differently in both groups. The mean shortening was 26 ms (SD = 35 ms), in CS, and 117 ms (SD = 122 ms) in CD. The GROUP x CONDITION x DIRECTION interaction was significant as well (F = 4.5, P<0.05). In CS during test trials we observed a similar shortening in both directions (28 ms, SD = 38 ms for right RM and 24 ms, SD = 32 ms for left RM), but in CD, we observed more shortening against dystonia (141 ms, SD = 128 ms) than towards dystonia (93 ms, SD = 111 ms).

### Velocity

There was a significant effect of group, which was due to CD patients being slower than CS (between subject factor: GROUP, F = 29.2, P<10^−5^). Velocity significantly increased similarly in both groups in test trials (within subject factor: CONDITION, F = 41.9, P<10^−6^). In CD, there was non-significant trend for faster RM against dystonia (46°/s, SD = 24°/s) than towards dystonia (33°/s, SD = 25°/s) (interaction: GROUP x CONDITION x DIRECTION, F = 3.3, P = 0.08).

### Relative amplitude

There was no effect of group on relative RM amplitude (between subject factor: GROUP, F = 2.7, P = 0.11). In patients, there was a significant effect of direction, with smaller amplitude towards dystonia (38.0, SD = 9.5%) than against dystonia (53.0, SD = 9.9%) (interaction: GROUP x DIRECTION, F = 14.5, P<0.001). In addition, there was a significant effect of condition, with increased relative amplitude in test with respect to control trials in both groups of subjects (within subject factor: CONDITION, F = 22.8, P<0.0001). The percentage increase was similar in both groups (2.7%, SD = 4.1% in CD and 4.6%, SD = 5.6% in CS).

### *FHA*

There was a significant effect of group on FHA, which was lower in CD than in CS (between subject factor: GROUP, F = 6.0, P<0.05). There was also a significant effect of direction (interaction: GROUP x DIRECTION, F = 5.6, P<0.05), which was due to patients reaching a larger FHA when rotating their head towards dystonia (55°, SD = 12°) than when they performed RM against dystonia (49°, SD = 11°). Test trials induced a significant increase in FHA in both groups of subjects (within subject factor: CONDITION, F = 24.3, P<0.0001). The relative increase was not significantly different between groups (5.1°, SD = 5.2° in CD vs. 4.0°, SD = 5.9° in CS), significant differences appeared when the limits of the BRM were considered. In test trials, the FHA was larger than the angle reached at the BRM assessment in 7 patients (44%) with RM towards dystonia and in 9 patients (56%) with RM against dystonia. These percentages were significantly larger than those expected with a probability range of 20%, as found in average in CS (44% vs. 20% probability: χ^2^ test = 3.8, P<0.05; 56% vs. 20% probability: χ^2^ test = 10.4, P<0.001).

### Data from EMG recordings

**Figure 5:**
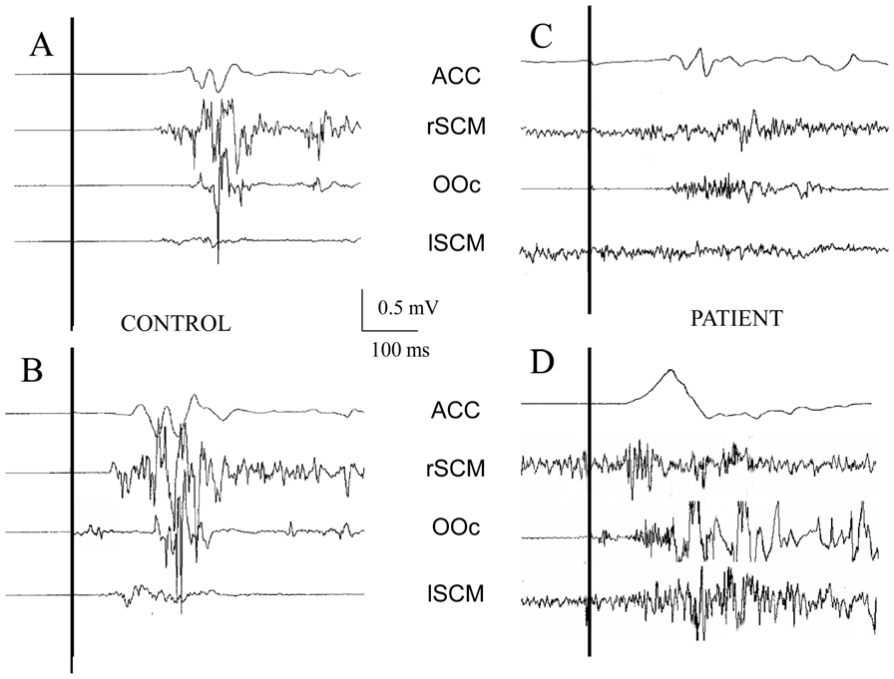
Representative examples of EMG and accelerometer recordings. Representative examples of EMG and accelerometer recordings from control (A and C) and test trials (B and D) in one control subject (CS) and one patient with cervical dystonia (CD).

EMG activity was recorded from the SCM in all control and test trials from CS and CD patients. However, the presence of continuous activity before IS, the absence of well- defined bursts in the agonist SCM and excessive movement artifacts prevented us from obtaining reliable measurements of latency and area in 5 patients and in 2 control subjects. Data from suitable recordings in the remaining 10 patients and 13 control subjects are summarized in Table 2. There was a large inter- and intra-individual variability in the data, which is reflected in the relatively large SD in both groups of subjects. The multivariate repeated analysis of variance showed no significant differences between CS and CD in the latency of the EMG activity for agonist and antagonist SCM, latency of the accelerometer signal and EMG area ratio between both SCM (between subjects factor GROUP: F = 0.5, p = 0.73). However, there were significant differences due to experimental condition within groups (within subject factor: CONDITION, F = 61.3, P<10^−8^). None of the interactions was significant. The significant differences found in CONDITION were due to a latency shorter in test than in control trials in the accelerometric signal (ANOVA, within subject factor: CONDITION, F = 200.6, P<10^−10^), the agonist SCM (F = 85.1, P<10^−7^) and the antagonist SCM (F = 96, P<10^−8^). No differences were found in the EMG area ratio (F = 0.8, P>0.05). Representative examples from one healthy subject and one patient are shown in Figure 5.

**Table 2:** Parametric data in healthy subjects and patients, separated into movements towards the dystonia side and those against the dystonia.

	Control subjects (N = 15)				Cervical dystonia patients (N = 15)			
	RM Left		RM Right		RM towards dystonia		RM against dystonia	
Trial	Control	Test	Control	Test	Control	Test	Control	Test
**RM onset latency (ms)**	211.4 (33.8)	127.7 (18.2)	207.5 (34.9)	120.3 (14.0)	217.9 (39.6)	133.5 (26.9)	215.4 (39.0)	124.8 (9.6)
**RM amplitude (%)**	40.6 (6.2)	43.6 (7.7)	39.9 (6.5)	42.5 (7.5)	35.7 (9.3)	40.2 (10.1)	50.0 (10.0)	55.2 (12.1)
**RM duration (ms)**	293.6 (57.3)	266.1 (36.7)	288.5 (59.9)	265.6 (42.1)	453.5 (170.2)	362.6 (103.7)	519.4 (197.8)	382.1 (110.6)
**RM velocity (deg/s)**	202.6 (56.4)	235.9 (65.2)	203.8 (51.6)	228.1 (50.1)	96.5 (32.7)	129.1 (41.4)	123.9 (52.2)	170.5 (50.4)
**FHA (deg)**	56.3 (9.6)	60.5 (12.0)	58.9 (11.4)	6201 (9.1)	53.2 (12.3)	57.8 (11.0)	45.9 (11.8)	51.2 (12.6)

FHA: Final head position angle, the angle between the inion-to-nasion line and the midline at the maximal angle reached in the RM.

RM: Rotational movements.

### Subjective rating of performance

CS did not report any difficulty in performing the RM in control or test trials. However, some of them reacted spontaneously after the first test trials, manifesting their surprise for having done the intended movement somehow involuntarily, without feeling the IS. A few patients made spontaneous comments after application of some test trials in line with those of CS, adding spontaneously that movement was easy to perform in trials containing the SAS. Patients rated their difficulty in performing RM at baseline with a mean of 7.3+/−2.0. Changes after the test were reported by 14 out of the 15 patients of the study (93.3%). In these patients, the mean rating in test trials was significantly higher than in control trials (9.2+/−1.1 vs 7.6+/−1.6; t-test; p<0.01). A total of 13 patients (92.8%) reported that movements were easier to perform in trials with SAS, while only one patient, a female with significant pain, reported more difficulties with performing RM in test (score of 8) than in control trials (score of 6).

## Discussion

The main findings of our study are the following: 1) Patients with CD had an abnormal performance of RM in baseline and control trials. The BRM was reduced in comparison to CS, with a maximum angle with respect to midline smaller against than towards the dystonic thrust. The RM velocity was lower, and the FHA was smaller in comparison to CS. 2) The presence of a SAS together with the sensory cue was accompanied with significant improvement of all RM measures in both groups. For RM duration, the effect was even larger in CD than in CS. In addition, patients were more likely to reach beyond their baseline maximal range of RM. 3) There was a higher degree of cocontraction between agonist and antagonist muscles in CD than in CS in control trials, but the differences did not reach statistical significance and 4) Patients felt that they performed movements easier when SAS accompanied the sensory cues.

Our findings suggest that CD patients are able to effectively prepare the motor structures engaged in performance of a voluntary movement in advance of its execution, with no difference with respect to healthy subjects. This was an unexpected finding since we hypothesized that patients would have difficulties in setting the appropriate preparation because of the various alterations described in sensorimotor integration 20,22. However, patients did not only well in the StartReact test but they did better than in the control condition. One possible explanation for these findings is that, with faster execution of the task in test trials, less time was left for eventual corruption of the prepared motor programme by interference of sensory inputs. It is known that ballistic movements can be executed with no sensory feedback [25]. However, the last part of the triphasic pattern of ballistic movements is indeed affected by sensory input [26], [27]. Since physiological abnormalities in dystonia are particularly involving the integration of sensory inputs into motor programmes [28], we could speculate that the ballistic nature of the movement performed in the StartReact test would have made it possible for the motor programme to be executed before sensory inputs could have intervened. Alternatively, the abnormal function of the basal ganglia circuitry that takes place in dystonic patients [29], [30] could have been bypassed by the SAS-related activation of the reticulospinal tract.

In fact, one of the theories explaining the StartReact phenomenon is that SAS is able to induce a faster execution of the preprogrammed movement by activating the brainstem reticular formation [31]. In a study testing the startle reaction in CD patients, Muller et al. (2003) found normal latency startle reactions, indicating that the reticular formation responded to loud auditory inputs [23]. The descending volley in the reticulospinal tract would activate the brainstem and spinal cord motoneurons involved in the requested task, which are already set at a high level of excitability during premovement motor preparation, and the task would be executed at a significant shorter latency than expected in case of premovement processing of the sensory cue [32]. However, recently Alibiglou and MacKinnon (2012) have gathered evidence that the motor cortex is indeed mediating at least part of the StartReact effect via a route that is faster than the conventional route followed by the sensory cue [15]. Whether the effects are mediated through the reticulospinal tract or through a cortical loop, our findings indicate an adequate level of subcortical motor preparation in CD patients.

Although EMG activity was recorded, we were unable to analyze with enough detail the traces from one third of our patients The CD patients exhibited large variability of EMG activity patterns in the SCM muscles. In some patients the SCM activity, either dystonic or compensatory, was already present at the time of IS, while in others there were bursts of activity with pauses that would not allow to clearly identify the onset of the activity related to the RM, in some patients the EMG activity was very poor. In the example given in Figure 5C, EMG activity is rather poor with no well-defined bursts even if the movement was indeed performed. It is possible that the EMG activity picked up with the electrodes attached over the SCM was generated by other not recorded synergistic muscles. Because of that, we preferred to use video-recordings from a high-speed camera for more reliable measurements of kinematic parameters. We considered that frame-by-frame video analysis would be a more suitable method of analysis of head RM in CD patients, even if the analysis was rather time consuming. Nevertheless, surface EMG provided relevant data when analyzed in the same individual in the two different conditions. It was clear in control trials that patients exhibited the already expected high level of co-contraction between agonist and antagonist muscles and that this did not change significantly in test trials. Therefore, even if SAS induced a normal acceleration of execution, its presence did not change the relationship between muscles. This is consistent with cocontraction being part of the motor programme, which is known not to be modified in the StartReact test.

Similar to previously published findings, based on other means of recording, we found normal RM latency in control trials in CD patients [33], [34]. However, CD patients performed RM much slower than CS [4]–[6], [26]. In order to avoid participation of compensatory mechanisms, the patients were asked not to correct the dystonic posture while waiting for the IS. This accounts for the finding of a larger SHA in CD than in CS. The deviation that patients had towards the side of the dystonia at Frame0 may account for the decreased RM amplitude and RM velocity when moving towards the dystonia. This, together with excessive co-contraction, may account for a limited FHA with RM to the side against dystonia, even though the amplitude of the RM to that side was not different from the mean values obtained in CS.

There was an improvement of performance in test trials in comparison to control trials. The mean angular velocity of RM towards and against the dystonia was faster in test than in control trials. The FHA increased more frequently beyond the maximal voluntary BRM in patients than in CS. Interestingly, patients reported spontaneously an improvement in their performance in trials with SAS with respect to pre-test values. The differences cannot be accounted for by learning or habituation since almost all patients were able to differentiate between trials with SAS and those without in regard to the effects on their performance. The improvement of motor performance by strong external stimuli and its clinical equivalent, the phenomenon of paradoxical kinesis, has been observed in Parkinson's disease patients [35], [36].

Our findings in CD patients would be in line with the results of other studies reporting improvement of motor performance with SAS using reaction time paradigms [11]–[14], [37]. Our patients reported a subjective improvement of their performance with SAS. We cannot give to this information any relevance apart from its subjective aspect. However, it indicates that patients performed indeed the task that they wanted to perform and they felt they performed it better than in control conditions. We can speculate on the possibility that the strong SAS allows CD patients to access additional motor pathways, or activate more effectively and consistently existing motor pathways, than they can do through will alone. The implication is that these circuits seem to be preserved in CD patients and accessible to indirect activation by startling acoustic stimuli, which could be potentially considered in therapeutic approaches.
